# Antimicrobial and Anticorrosion Activity of a Novel Composite Biocide against Mixed Bacterial Strains in Taiwanese Marine Environments

**DOI:** 10.3390/ma14206156

**Published:** 2021-10-17

**Authors:** Soul-Yi Chang, Shih-Yen Huang, Yu-Ren Chu, Shun-Yi Jian, Kai-Yin Lo, Yueh-Lien Lee

**Affiliations:** 1 Department of Engineering Science and Ocean Engineering, National Taiwan University, Taipei City 106, Taiwan; r06525074@ntu.edu.tw (S.-Y.C.); r08525129@ntu.edu.tw (S.-Y.H.); zeeve.y.chu@gmail.com (Y.-R.C.); 2 Department of Chemical & Materials Engineering, Chung Cheng Institute of Technology, National Defense University, Taoyuan City 335, Taiwan; ftvko@yahoo.com.tw; 3 Department of Agricultural Chemistry, National Taiwan University, Taipei City 106, Taiwan

**Keywords:** biofouling, composite biocides, corrosion, electrochemical impedance spectroscopy, confocal laser scanning microscopy

## Abstract

Taiwan is an island with a humid subtropical climate. The relatively warm seawater results in biofouling of the surfaces of marine facilities. Biocide application is a common practice for combating and eliminating adhesive fouling. However, a single type of biocide may have limited antimicrobial effects due to the relatively high microbial diversity in marine environments. Therefore, applying a mixture of various biocides may be necessary. In this study, the antimicrobial and anticorrosion properties of a newly designed composite biocide, namely a combination of thymol and benzyldimethyldodecylammonium chloride, were investigated by applying the biocide to 304 stainless steel substrates immersed in inocula containing bacterial strains from Tamsui and Zuoying harbors. The ability of 3TB and 5TB treatments to prevent sessile cells and biofilm formation on the 304 stainless steel coupon surface was determined through scanning electron microscopy investigation. In addition, confocal laser scanning microscopy indicated that the 5TB treatment achieved a greater bactericidal effect in both the Tamsui and Zuoying inocula. Moreover, electrochemical impedance spectroscopy revealed that the diameter of the Nyquist semicircle was almost completely unaffected by Tamsui or Zuoying under the 5TB treatment. Through these assessments of antimicrobial activity and corrosion resistance, 5TB treatment was demonstrated to have superior bactericidal activity against mixed strains in both southern and northern Taiwanese marine environments.

## 1. Introduction

Corrosion is a common phenomenon that leads to the failure of engineering materials in marine environments. The economic losses resulting from microbiologically influenced corrosion constitute approximately 20% of all losses attributable to corrosion, and the cost of microbiologically influenced corrosion is equivalent to 2% of the gross national product of developed countries [1,2]. Thus, the effect of microbiologically influenced corrosion should not be overlooked. The failure of materials due to microbiologically influenced corrosion is mainly attributable to the formation of biofilms containing mixed microorganisms. Microbiologically influenced corrosion is an electrochemical process involving interactions among microorganisms, metals, and the corrosive environment. Various studies have focused on the aerobic and anaerobic microbial corrosion mechanisms by single species of bacteria, including sulfate-reducing bacteria (SRB) [3,4,5], iron-oxidizing bacteria (IOB) [6,7,8], nitrate-reducing bacteria (NRB) [9,10], and methanogenic bacteria [11,12], among other types [13,14,15]. Numerous researchers have made progress in studying the corrosion behavior and interactions in a mixed system of SRB/IOB [16,17] and SRB/NRB [18,19]. The literature indicates that the synergistic action of mixed bacterial strains exerts a strong corrosive effect on metals. Furthermore, biofilms formed by mixed strains are relatively loose and provide a channel for oxygen transmission, and mixed strains produce corrosive metabolites such as sulfides, phosphides, and other acids, which further exacerbate localized corrosion.

To control biocorrosion, various techniques have been employed, including protective coatings [20], corrosion inhibitors [21], cathodic production [22,23], biological approaches [24], and biocides [25,26]. Among these methods, all of which are based on the inhibition of bacterial growth and excessive biofilm formation, biocides are the most commonly used. However, many traditional biocides, such as tributyltin and heavy metal compounds, are environmentally hazardous and may exert adverse effects on immune responses in aquatic animals due to the release of disinfectant byproducts. Herein, the use of less toxic or environmentally friendly compounds for treating microbiologically influenced corrosion is considered.

Benzyldimethyldodecylammonium chloride (BDMDAC) consists of quaternary ammonium compounds (QACs) and benzalkonium chloride (BAC), a type of cationic surfactant comprising two components [27]. QAC antimicrobial activity has been attributed to their long alkyl chains, which promote electrostatic interactions with negatively charged areas on cell membranes [28,29]. Thus, QAC cations and anionic groups of membrane polymers can be cross-linked to disrupt bacterial cells. In our previous study, the antimicrobial properties of BDMDAC in anaerobic conditions against *Dulsulfovibrio desulfuricans* were investigated. We previously observed that the application of 25 ppm BDMDAC achieved satisfactory results against microbial corrosion by *D. desulfuricans* [30]. According to their respective material safety datasheets (Alfa Aesar, Ward Hill, MA, USA), the medial lethal dose (LD_50_) of BDMDAC indicates it is less toxic than tributyltin (LD_50_ of BDMDAC: 400 mg/kg in rats; LD_50_ of tributyltin: 132 mg/kg in rats), but the toxicity of BDMDAC continues to call into question its use. Furthermore, a single BDMDAC may exert limited antimicrobial effects due to the relatively high microbial diversity of marine environments. Therefore, the application of a mixture of various biocides may be required in local seawater.

Thymol is a monoterpenoid phenol derivative of cymene found in thyme essential oil. The majority of essential oils are classified as generally recognized as safe substances by the US Food and Drug Administration [31]. However, thymol is easily degraded in the marine environment because of its chemical structure [32]. Owing to its lipophilic properties, thymol exhibits inhibitory activity against sessile organisms and biofilm formation, which can reduce the stability of cell membranes and interfere with the structure of the phospholipid bilayer [33,34]. Notably, the LD_50_ of thymol (LD_50_ of thymol: 980 mg/kg in rats) is two to three times higher than that of BDMDAC, indicating thymol’s potential for use in environmentally friendly antibiofouling strategies.

The goal of this study was to evaluate the antimicrobial properties of composite biocides by examining the effect of a mixture of thymol and BDMDAC as a composite biocide against *Shewanella* and *Vibrio*. This study is the first to report the biocidal activity of a composite biocide against representative bacterial strains in the coastal waters of Taiwan. The usage and concentrations of each compound in the composite biocide were determined by their minimum inhibitory concentrations (MICs). The influence of this composite biocide and mixed field strains on the corrosion behavior of 304 stainless steel (304 SS) was systematically analyzed using polarization curves, scanning electron microscopy (SEM), and confocal laser scanning microscopy (CLSM).

## 2. Materials and Methods

### 2.1. Sample Preparation

The square 304 SS coupons (30 mm × 30 mm × 1 mm) were prepared. The surfaces were abraded with silicon carbide papers of varying grades (80, 400, 800, and 1200 grit), rinsed with deionized water, and ultrasonically washed with ethanol. All coupons were placed in an experimental laminar airflow chamber and sterilized under an ultraviolet lamp (Genprice Inc., San Jose, CA, USA) for 60 min before incubation.

### 2.2. Mixed Strains and Culture Media

The study sites were the harbors of Tamsui and Zuoying (25°10′59.1″ N, 121°24′41.7″ E and 22°41′22.4″ N, 120°16′25.9″ E), which are located in northern and southern Taiwan, respectively. Mixed strains were obtained separately from these harbors and grown aerobically in Marine Broth 2216 (Zobell Marine Broth: NaCl, 19.45 g/L; MgCl_2_, 5.9 g/L; MgSO_4_, 3.24 g/L; CaCl_2_, 1.8 g/L; KCl, 0.55 g/L; NaHPO_3_, 0.16 g/L; ferric citrate, 0.1 g/L; KBr, 0.08 g/L; peptone, 5.0 g/L; yeast extract, 1.0 g/L). Prior to each test, the mixed strains were inoculated with broth culture for 24 h, and bacterial cultivation was performed at 30 °C.

### 2.3. Mixed Strain Purification

**Figure 1:**
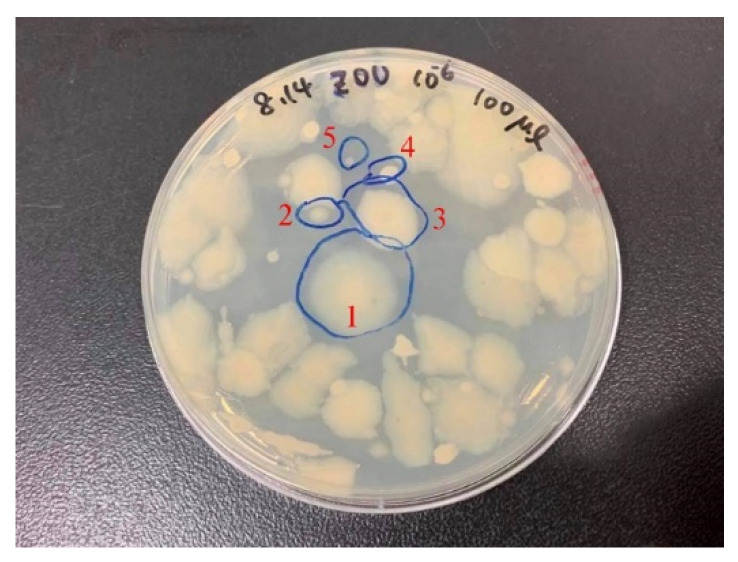
Colony morphology of Zuoying inocula on the marine broth agar.

Agar dilution is the most commonly used method to purify bacteria and thus identify representative species. In the present study, a 1 mL aliquot of a bacterial suspension was transferred from the water sample to a 1.5 mL microcentrifuge tube and then diluted with sterile water to produce solutions of varying concentrations. Each solution was spread on a separate marine broth agar plate. The inoculated plates were incubated for 24 h at 30 °C. The colony morphologies after each purification step are graphically presented in Figure 1.

### 2.4. Polymerase Chain Reaction and Strain Identification

For strain identification, we amplified the genomic DNA extracted from isolated colonies using primers. Polymerase chain reaction (PCR) was performed in a final volume of 25 μL containing a template, 1.25 units of *YEA*taq II DNA polymerase (Yeastern Biotech, Taipei, Taiwan), 10× buffer, 0.2 μM primers, 0.2 mM deoxynucleoside triphosphate, and distilled water. The thermal cycling conditions were as follows: an initial denaturing step at 95 °C for 5 min, followed by 30 cycles of denaturation at 95 °C for 30 s, annealing at 55 °C for 30 s, extension at 72 °C for 30 s or 120 s, and then a final extension at 72 °C for 10 min. The 345/346 and 347/348 primers were used to amplify 400 base pairs (bp) and 1200 bp of 16S rDNA, respectively. The PCR products were subsequently purified using the DNA Clean & Concentrator kit (Zymo Research, Irvine, CA, USA), quantified with the Qubit dsDNA HS Assay Kit (Thermo Fisher Scientific, Waltham, MA, USA), and sequenced at the Institute of Biotechnology at National Taiwan University. The Basic Local Alignment Search Tool (BLAST) was used to align sequences to the Nucleotide database maintained by the US National Center for Biotechnology Information [35]. Strain identification was performed after sequence alignment.

### 2.5. MICs of Composite Biocides

The inhibition of bacterial growth and biofilm formation are the key aspects of treating microbiologically influenced corrosion. MIC determination, an essential measure of the antimicrobial performance of composite biocides, was conducted herein, as described by Wiegand and Balouiri [36,37], with minor modifications. Broth dilution, a basic antimicrobial susceptibility testing method, involved preparing dilutions of antimicrobial agents in a liquid growth medium with lower volumes on a 96-well microtiter plate. Each concentration was detected with three replicates. Each well was inoculated with microbial inoculum prepared in the same medium, and the bacterial suspension was diluted according to the 0.5 McFarland standard, corresponding to an optical density of 0.1 at 600 nm (O.D._600_). McFarland standards [38] are employed as a reference for examining the approximate number of bacteria in a liquid suspension according to the turbidity of the suspension, which in the present study was approximately 1.5 × 10^9^ CFU/mL. In each well, various concentrations of composite biocides were mixed separately. The 96-well plate was next incubated without agitation for 36 h at 30 °C. The absorbance of the inocula was measured every 2 h on an enzyme-linked immunoassay reader (Infinite 200 Pro, Tecan, Switzerland). The results are expressed as the MICs of composite biocides against the mixed microorganisms. All the MIC measurements were conducted at least three times to confirm the reproducibility of the results.

### 2.6. Crystal Violet Assay for Biofilm Quantification

Crystal violet (CV) can be used to stain the nuclei of adherent cells. Herein, CV staining was performed to quantify the accumulation of biomass in tubes and evaluate the antimicrobial activity of the composite biocides. First, the absorbance of the bacterial suspensions was adjusted to an O.D._600_ of 0.1 with fresh medium, and 2 mL of culture was transferred to each tube. Various concentrations of composite biocides were added, and the tubes were incubated without agitation for 5 days at 30 °C to allow biofilm formation. Subsequently, the tubes were gently rinsed twice with deionized water, after which 2 mL of CV was used to stain the cells for 15 min at room temperature. The tubes were then washed twice and dried. Finally, 2 mL of 30% acetic acid was added to each tube. Antimicrobial activity was estimated by measuring the O.D._550_ value.

### 2.7. MIC Immersion and Electrochemical Measurements

Five immersion conditions were applied before the polarization curve measurement: (1) 304 SS coupon in the culture medium (labeled as blank), (2) 304 SS coupon in the Tamsui or Zuoying inocula (labeled as Tamsui or Zuoying), (3) 304 SS coupon in the bacterial culture medium and composite biocides at the MIC (labeled as TB), (4) 304 SS coupon in the bacterial culture medium and composite biocides at triple the MIC (labeled as 3TB), and (5) 304 SS coupon in the bacterial culture medium and composite biocides at five times the MIC (labeled as 5TB).

Electrochemical impedance spectroscopy (EIS) was performed in a 3.5 wt % NaCl solution by using a potentiostat (Reference 600, Gamry Instruments, Warminster, PA, USA). The electrochemical cell was composed of a 304 SS coupon, platinum plate, and saturated calomel electrode as the working, counter, and reference electrodes, respectively. The testing area on the 304 SS coupon for electrochemical measurements was 7 cm^2^. To obtain the electrochemical responses generated from the pure surface of corroded 304 SS, all biofilms were stripped from the surface of the 304 SS coupons in an ethanol ultrasonic bath before EIS. Moreover, prior to EIS, the coupons were immersed in a NaCl solution for 1800 s to ensure that a stable open circuit potential was reached. The EIS measurements were then recorded at an open circuit potential in the frequency range of 10^5^ to 10^−2^ Hz, employing an alternating current amplitude of 10 mV.

### 2.8. Surface Morphology Examination

After immersion in working solutions over varying durations, the surface morphologies of 304 SS coupons were examined under a scanning electron microscope (JSM-6510, JEOL, Tokyo, Japan). The chemical compositions of the biofilms were analyzed using an energy-dispersive X-ray spectrometer (INCA x-act, Oxford Analytical Instruments, Abington, UK). The 304 SS coupons that had undergone 12 or 336 h of immersion in various working solutions were subjected to SEM. Before the procedure, the coupons were first removed from the solutions and fixed with 2.5% (*v*/*v*) glutaraldehyde solution for 20 min. They were then sequentially dehydrated with ethanol solutions (25%, 50%, 75%, and 99%) and air dried.

### 2.9. Biofilm Characterization

For biofilm characterization, the 304 SS coupons were immersed under the five aforementioned conditions for 12 or 336 h. The coupons were subsequently rinsed twice with a phosphate-buffered saline (pH 7.0) solution. The live and dead bacteria in the biofilm were imaged after staining with the LIVE/DEAD BacLight Bacterial Viability Kit (Thermo Fisher Scientific, Eugene, OR, USA) containing SYTO 9 (3.34 mM in dimethyl sulfoxide) and propidium iodide (20 mM in dimethyl sulfoxide). Fluorochromes were excited using a tunable laser with a photomultiplier tube filter, and a GaASP detector was employed. The coupons were placed in the devices with coverslips, mounting oil, and clear nail polish prior to CLSC (LSM780, Carl Zeiss, Jena, Germany). Z-stacking and images captured with the LSM780 camera were used to determine the thickness of the biofilm. Optical sections approximately 0.2 μm in height were collected from the bottom to the top of the biofilm, after which the entire biofilm architecture was visualized.

## 3. Results

### 3.1. Strain Purification and Classification

**Figure 2:**
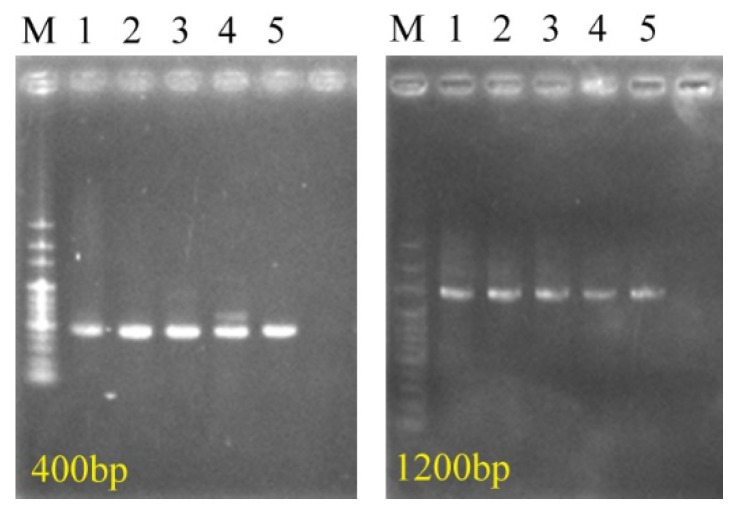
16S rDNA from the Zuoying isolates amplified through PCR. M: Marker; Lanes 1, 2, 3, 4, and 5: ISZ1, ISZ2, ISZ3, ISZ4, and ISZ5, respectively.

With reference to a study [37], the agar dilution method and PCR were employed for strain purification and classification. In our previous study, we examined mixed strains collected from Tamsui harbor [39]. Figure 1 displays the colony morphology of mixed strains incubated overnight on the marine broth agar. Five representative strains were identified, as indicated by the areas circled in the figure. Figure 2 presents the PCR products amplified from 16S rDNA from the Zuoying strains. The PCR products were purified and sequenced. The sequences were aligned to the Nucleotide database with BLAST. Table 1 shows the representative strains from Tamsui and Zuoying harbors. The majority of the isolated strains from the Zuoying inocula were Gram-negative bacteria, characterized by their thin peptidoglycan cell wall and outer membrane. In the Zuoying inocula, *Vibrio* was the main genus found.

### 3.2. Determination of the MICs of Composite Biocides

To determine the MICs of the composite biocides, the O.D._600_ of the Tamsui and Zuoying inocula were measured separately over 36 h. Moreover, the antimicrobial activity of different concentrations of composite biocides were evaluated against two inocula. The testing concentrations of thymol or BDMDAC ranged from 0 to 60 mg/L.

**Figure 3:**
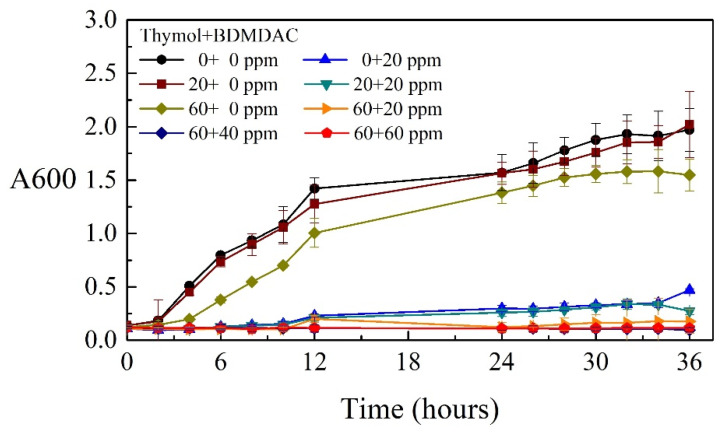
Microbial growth curves of varying concentrations of composite biocides applied to the Tamsui inocula.

**Figure 4:**
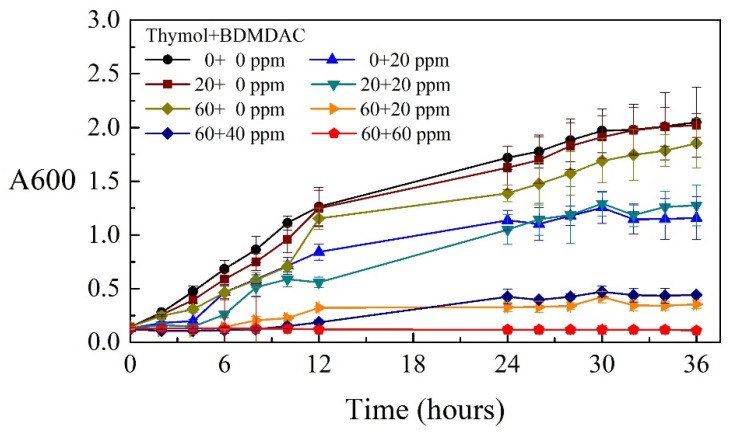
Microbial growth curves of varying concentrations of composite biocides applied to the Zuoying inocula.

Figure 3 and Figure 4 display the growth curves of the Tamsui and Zuoying inocula over 36 h. Figure 3 presents the antimicrobial activity of composite biocides against the Tamsui inocula. Treatment with thymol only (20 and 60 mg/L) yielded weak antimicrobial effects; these effects were superior under treatment with 20 mg/L BDMDAC. Although the combined use of 20 mg/L BDMDAC and 20 mg/L thymol did not enhance antimicrobial activity, the application of 60 mg/L thymol resulted in relatively high antimicrobial activity and had similar effects as did higher concentrations of composite biocides (i.e., 60 mg/L thymol and 40 mg/L BDMDAC; 60 mg/L thymol and 60 mg/L BDMDAC). A similar tendency in antimicrobial effects against the Zuoying inocula was observed (Figure 4). Specifically, treatment with thymol alone (20 and 60 mg/L) resulted in weak antimicrobial activities. Treatment with 20 mg/L BDMDAC or with 20 mg/L thymol was less effective against the Zuoying inocula than against the Tamsui inocula. However, the combined application of 20 mg/L BDMDAC with 60 mg/L thymol promoted the inhibition of bacterial growth. Thus, the MIC of composite biocides against mixed strains isolated from the study sites was determined to be 60 mg/L thymol + 20 mg/L BDMDAC. The combination of 60 mg/L thymol and 20 mg/L BDMDAC is presented as TB in the following sections.

### 3.3. Effect of Composite Biocides on Biofilm Formation: CV Staining

**Figure 5:**
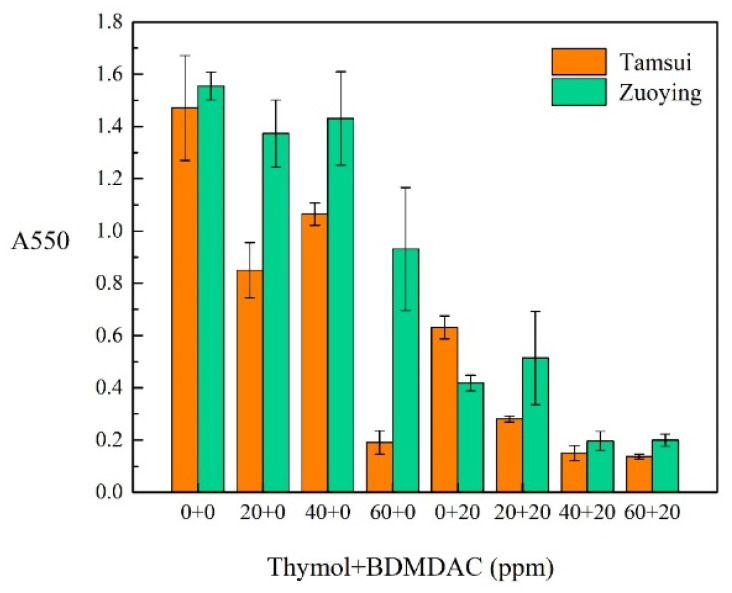
Treatments of varying concentrations of biocides to inhibit biofilm formation in the Tamsui and Zuoying inocula.

The inhibition of biofilm formation was quantified through CV staining. As shown in Figure 5, the antibiofilm activity of varying concentrations of biocides (0, 20, 40, and 60 mg/L thymol + 0 and 20 mg/L BDMDAC) against Tamsui and Zuoying inocula was examined. Each tube was incubated for 5 days at 30 °C, allowing biofilm to form at the air–liquid interface. Treatment with 60 mg/L thymol resulted in favorable antibiofilm activity in the Tamsui inocula but not in the Zuoying inocula. The combination of 40 mg/L thymol and 20 mg/L BDMDAC was effective in inhibiting biofilm formation in both the Tamsui and Zuoying samples.

### 3.4. Surface Morphology and Characterization

**Figure 6:**
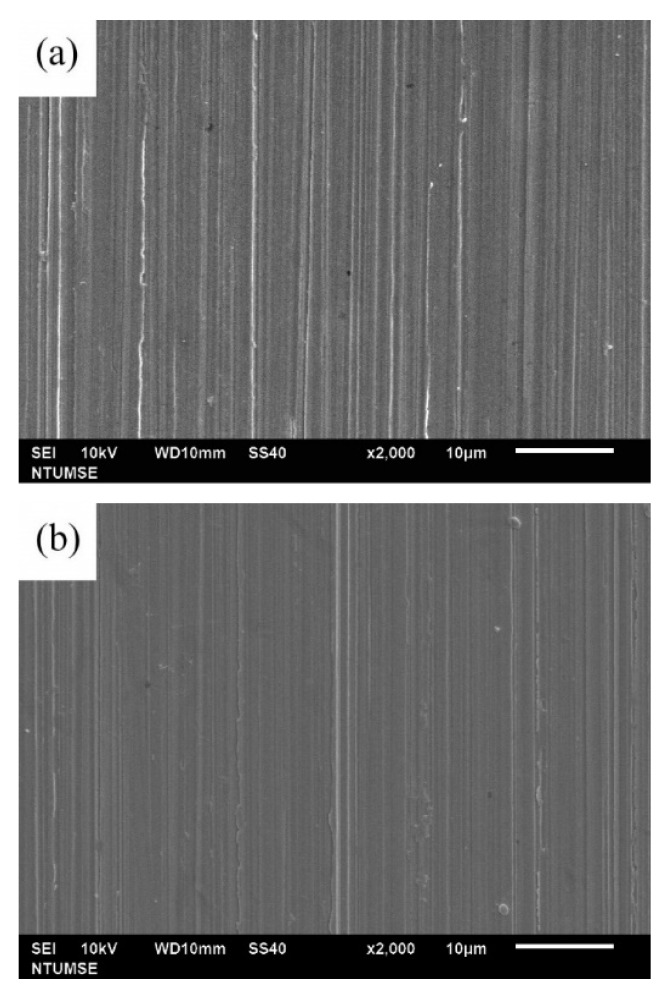
SEM images of the 304 SS surface after (**a**) 12 h and (**b**) 336 h of exposure to the uninoculated culture medium.

**Figure 7:**
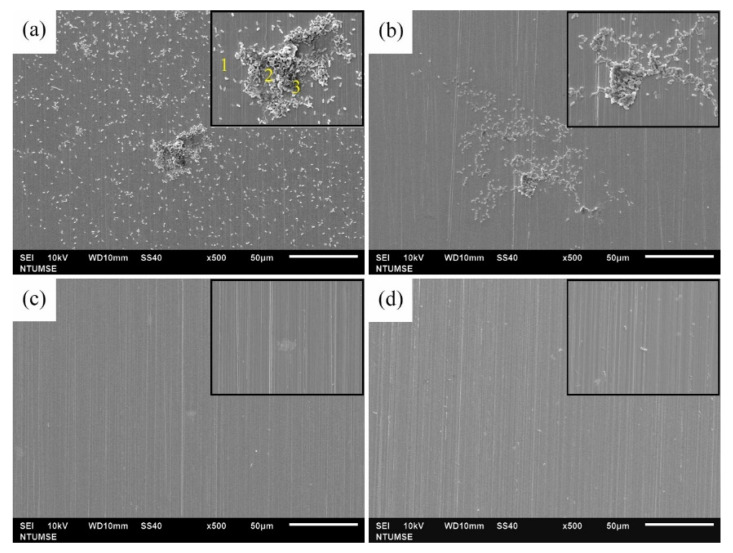
SEM images of the 304 SS surface after 12 h of exposure to (**a**) the uninoculated Tamsui medium, (**b**) TB, (**c**) 3TB, and (**d**) 5TB, presenting enlargements of areas with biofilm formation.

**Figure 8:**
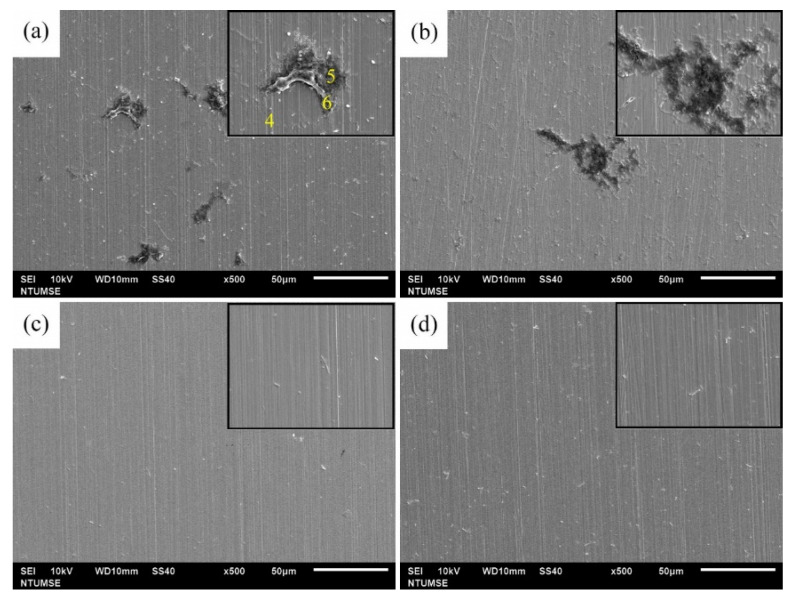
SEM images of the 304 SS surface after 336 h of exposure to (**a**) the uninoculated Tamsui medium, (**b**) TB, (**c**) 3TB, and (**d**) 5TB, presenting enlargements of areas with biofilm formation.

**Figure 9:**
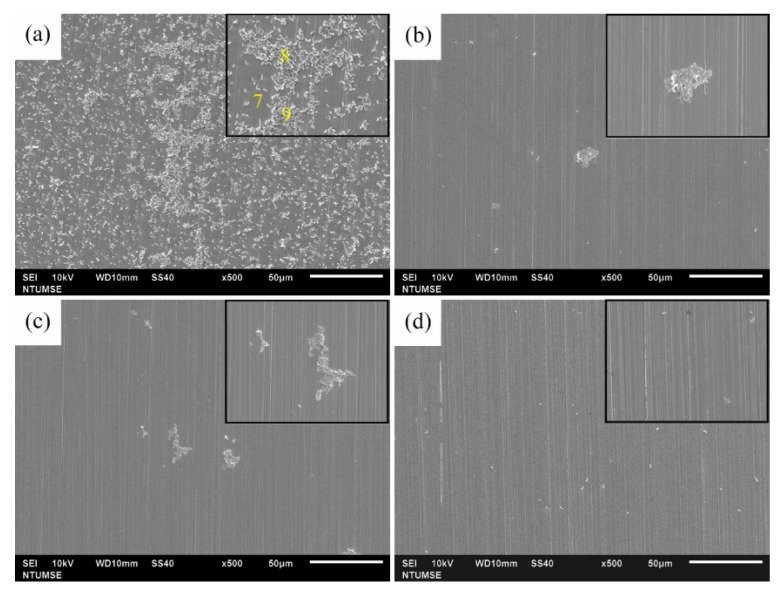
SEM images of the 304 SS surface after 12 h of exposure to (**a**) the uninoculated Zuoying medium, (**b**) TB, (**c**) 3TB, and (**d**) 5TB, presenting enlargements of areas with biofilm formation.

**Figure 10:**
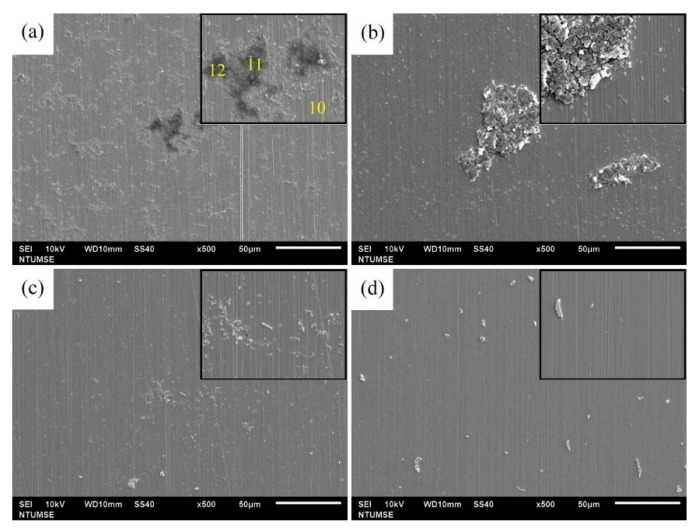
SEM images of 304 SS surface after 336 h of exposure in (**a**) the Zuoying inocula medium without treatment, (**b**) TB, (**c**) 3TB, and (**d**) 5TB, presenting enlargements of areas with biofilm formation.

The surface morphologies of 304 SS coupons after 12 and 336 h of immersion in Tamsui and Zuoying inocula with the application of composite biocides at varying concentrations were characterized through SEM (Figure 6, Figure 7, Figure 8, Figure 9 and Figure 10). For comparison, SEM images of the 304 SS coupons immersed in the uninoculated culture medium are also presented. As shown in Figure 6a,b, only ground grooves caused by the polishing process were distinguishable on the surface of the 304 SS coupons after 336 h of immersion in the culture medium. However, a large number of rod-shaped sessile cells, either isolated or grouped in small clusters, were observable on the coupons immersed in the uninoculated Tamsui and Zuoying inocula (Figure 7a and Figure 9a). Furthermore, deposits of a dense, lumpy substance were noted on the surface of the coupons subjected to 336 h of immersion (Figure 8a and Figure 10a). As shown in Table 2, energy-dispersive X-ray spectroscopy (EDS) revealed that this substance was mainly composed of Fe, Cr, Ni, O, S, and P. Iron oxide, among other oxides, is a possible source of oxygen. S can be traced to the SRB in the inocula; this may constitute evidence of the formation of FeS in the biofilm [5,30]. The presence of P is related to the sodium phosphate (Na_2_PO_4_) in the culture medium. The phosphate (PO_4_^3−^) in the Na_2_PO_4_ that accumulates on steel substrates when the medium contains sulfides and when the pH of the surrounding environment is less than 7 tends to be reduced to iron phosphide (Fe_2_P), especially in anaerobic environments [40]. Thus, the EDS results were indicative of bacterial adhesion and biofilm development in both the Tamsui and Zuoying inocula, regardless of the significant reduction in the number of sessile cells found on the coupons’ surfaces after immersion in the inocula with TB application. The sessile cells in Figure 7b and Figure 9b suggest that the efficiency of the composite biocide at the MIC was limited. By contrast, few sessile cells, if any, were detected on the coupons immersed in the Tamsui or Zuoying inocula under 3TB and 5TB treatment, as shown in Figure 8c,d and Figure 10c,d. These findings suggest that a composite biocide with three or five times the MIC was required to minimize or prevent bacterial adhesion and biofilm formation on the coupons immersed in the Tamsui and Zuoying inocula, respectively.

### 3.5. Characterization of Biofilm and Antimicrobial Activity

**Figure 11:**
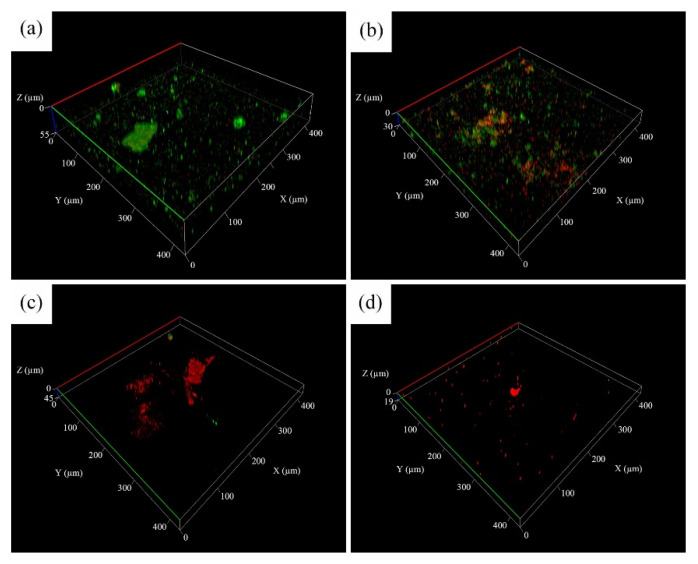
CLSM images of Tamsui inocula incubated for 336 h with varying concentrations of composite biocides under (**a**) no treatment, (**b**) TB, (**c**) 3TB, and (**d**) 5TB.

**Figure 12:**
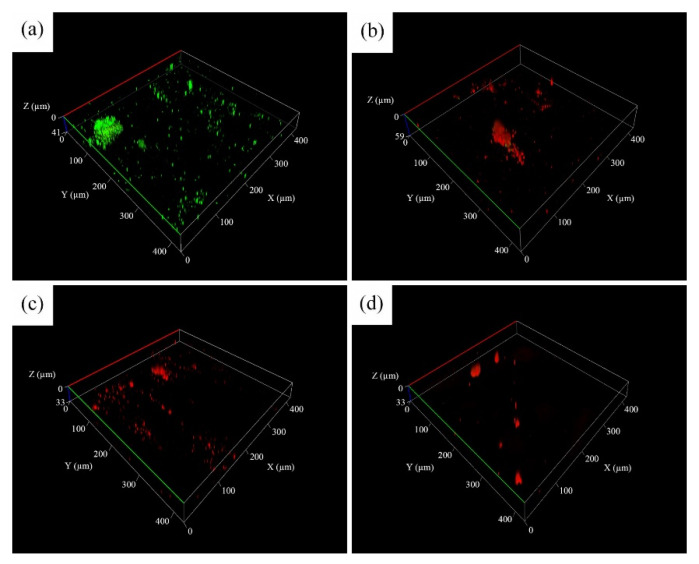
CLSM images of Zuoying inocula incubated for 336 h with varying concentrations of composite biocides under (**a**) no treatment, (**b**) TB, (**c**) 3TB, and (**d**) 5TB.

Although the attachment of sessile cells and biofilms is easily observable, differentiating between live and dead sessile cells in biofilms through SEM micrographs of surface morphology is challenging. Thus, CLSM was employed to examine the status of sessile cells deposited on the surface of 304 SS coupons after the immersion tests. As presented in Figure 11a and Figure 12a, a large number of live cells (green) were noted on the coupons immersed in the uninoculated Tamsui and Zuoying inocula. By contrast, dead cells (red) were mainly observed when various composite biocides were applied to the inocula (Figure 11b–d and Figure 12b–d). However, the green dots in Figure 11b,c and Figure 12b suggest that the TB and 3TB treatments resulted in inadequate antimicrobial effects. Compared with the TB- and 3TB-treated coupons, the 5TB-treated coupons exhibited significantly reduced bacterial cell populations. In addition, only dead cells were noted on the 304 SS coupons immersed for 336 h, whether in the Tamsui or Zuoying inocula (Figure 11d and Figure 12d). These findings suggest that the attachment of cells to the surface of 304 SS coupons can be strongly inhibited by both cell deactivation and cell death under the 5TB treatment [41].

### 3.6. EIS

**Figure 13:**
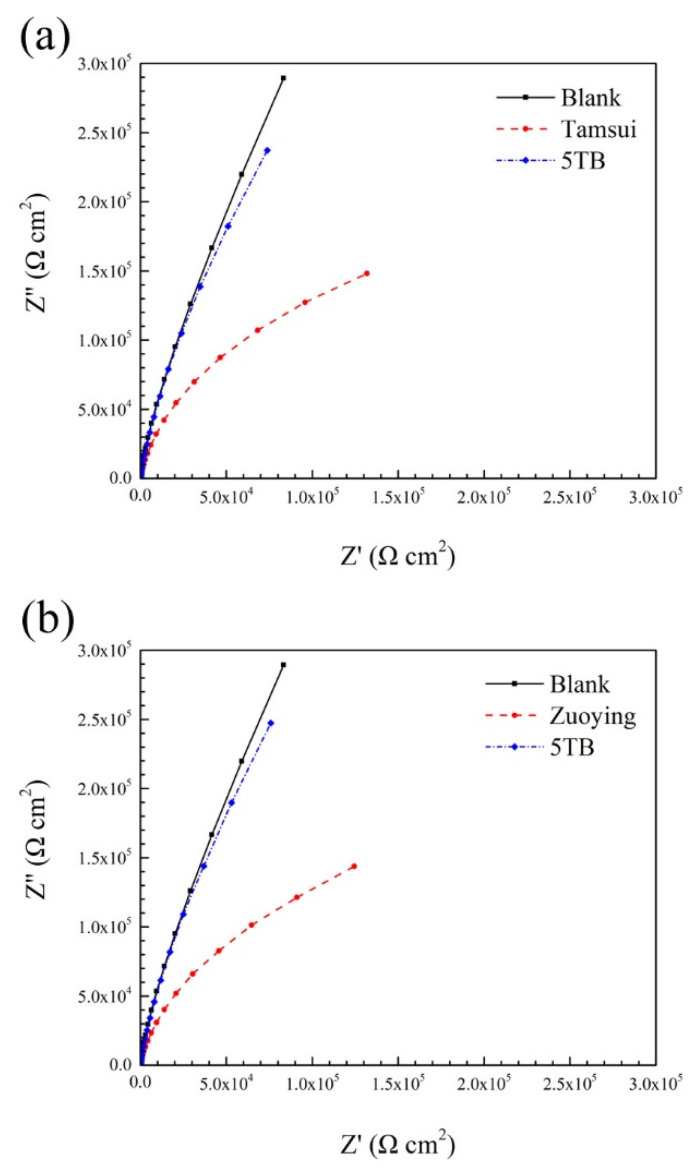
Nyquist plots of 304 SS coupons following 168 h of immersion in the (**a**) Tamsui inocula and (**b**) Zuoying inocula.

EIS is a well-known, nondestructive method for evaluating the electrochemical properties of metal surfaces. Nyquist plots of coupons immersed in various media over 168 h were generated (Figure 13). All the coupons exhibited similar features, namely incomplete semicircles. However, the dimensions of the semicircles differed by coupon. In general, the diameter of semicircles in Nyquist plots reflects the corrosion behavior at the interface of the film–electrolyte interface, with larger diameters typically indicating higher corrosion resistance [42]. The diameter of the semicircles corresponding to the Tamsui and Zuoying inocula were smaller than that of the semicircle corresponding to the uninoculated culture medium. This result suggests that the Tamsui and Zuoying inocula destroyed the passive properties of 304 SS over the 168 h of immersion. By contrast, the diameter of the semicircle was almost completely unaffected by the Tamsui and Zuoying inocula under the 5TB treatment. This means the application of composite biocides at five times the MIC was effective in killing most bacterial cells, thereby preventing the coupons from exhibiting microbiological degradation or deterioration with regard to the passive properties of the steel surface.

## 4. Discussion

Corrosive bacteria derive nutrition from various sources and are able to oxidize numerous carbon sources utilized as electron donors, including hydrocarbons, methanol, ethanol, acetate, propionate, butyrate, and sugar [43]. Among these model organisms, sulfate SRB primarily contribute to souring in oil field pipelines [44], and the synergistic effects of SRB and CO_2_ under a soil layer promote the corrosion of X52 steel [45]. In the present study, *Shewanella* and *Vibrio* were identified as the two major bacterial genera, and these strains were dissimilatory metal-reducing microorganisms (Table 1). Studies have demonstrated that *Shewanella* and *Vibrio* are dominant genera in seawater and that SRB are not abundant in transported seawater [46,47]. As iron-reducing bacteria, *Shewanella* spp. can dissolve protective ferric oxide layers (Fe^3+^) for anaerobic respiration [47]. *Vibrio* spp. are fast-growing facultative anaerobic bacteria, as indicated by a doubling time at 37 °C of 9.8 min. The extracellular polymeric substances around the bacterial cells and, as revealed through CLSM, the adhesion of live bacterial cells on the coupons’ surfaces indicate that the bacterial cells enhanced the corrosion rate of the 304 SS. A developmental process involving microbiologically influenced corrosion and mixed microorganisms has been reported [17,47]. Bacteria colonize metal surfaces, and oxygen is depleted through aerobic respiration. Localized anodes and cathodes accelerate the corrosion rate. As oxygen is depleted, bacteria respire through fermentation, which provides SRB with a favorable growth environment. The interaction between aerobic and anaerobic bacteria promotes the corrosion of metal materials. These corrosion mechanisms may allow aggressive species such as sulfides to affect metals [48,49].

The mechanism of action of biocides, including QACs [50,51] and essential oils [52,53,54], has been investigated. QACs with chain lengths between C12 and C16 have maximal efficacy [55]. These permanent positive charges enable such QACs to bind readily to microbes’ negatively charged membranes, causing cell leakage and membrane damage [50]. Thymol is hydrophobic and prone to interacting with the outer membrane of Gram-negative bacteria, causing a fluidifying effect, triggering the release of lipopolysaccharides, and increasing the permeability of the cytoplasmic membrane to ATP. Thus, thymol hinders the energy-generating processes of Gram-negative bacteria and reduces their recovery ability [56,57]. The MIC of biocides can be reduced through a synergistic effect. Essential oils have various antimicrobial effects [56]. The synergistic antimicrobial activity achieved through a combination of QACs and Cu^2+^ is effective in controlling biofilm formation [58]. The present study is the first to report the combined effects of thymol and BDMDAC. Treatments with three concentrations of composite biocides (thymol + BDMDAC) led to synergistic effects. The MIC of thymol against Gram-negative bacteria was reduced from 256–500 mg/L to 60 mg/L through synergistic action with BDMDAC [53,59]. Furthermore, EIS demonstrated that the composite biocides, in certain concentrations, were effective in protecting the stainless steel substrate against microorganisms.

After 336 h of incubation, the microorganisms treated with the composite biocides at the MIC were still able to grow. However, the exponential phase, during which cell doubling occurs, was longer. CLSM indicated that this concentration of composite biocides killed most cells (red dots) and delayed biofilm development. Approximately 50% of live cells (green dots) were found on the coupons’ surfaces. The application of an agent at the MIC usually means that bacterial growth is prevented, and the exponential phase is prolonged. The minimum bactericidal concentration (MBC) is the lowest concentration of an antimicrobial agent required to kill ≥99.9% of bacteria in starting inocula upon subculture (i.e., a 3-log_10_ reduction in colony-forming units per milliliter) [60]. Bactericidal activity can be defined as an MBC to MIC ratio of ≤4 [61]. Thus, we increased the composite biocide concentration to the MBC—approximately triple the MIC (3TB)—to ensure that 99.9% of bacteria could be killed and that bacterial cell growth could be inhibited. Thus, the growth of marine microorganisms was controlled over the short term. For long-term control, higher concentrations are required to eliminate bacterial populations. Consequently, five times the MIC (5TB) was selected as a composite biocide concentration.

## 5. Conclusions

In the present analysis of mixed bacterial strains isolated from the Tamsui and Zuoying harbors in Taiwan, *Shewanella* and *Vibrio* were identified as the two dominant species. The combination of phenolic and QACs exerted strong antimicrobial effects and was effective in slowing the corrosion rate of the 304 SS coupons. SEM and CLSM revealed that the composite biocides reduced the adhesion of sessile cells and biofilm. Through a synergistic effect, the dose of thymol and BDMDAC used to reduce bacterial growth in the combination treatment was considerably lower than those in the individual treatments. The 5TB treatment had favorable antimicrobial and anticorrosion effects in both southern and northern Taiwanese marine environments.

## Figures and Tables

**Table 1:** Representative strains of Microorganisms from Tamsui and Zuoying harbors.

Tamsui Strains	Microorganism
IST1	*Oceanimonas doudoroffii* [39]
IST2	*Shewanella algae* [39]
	*Shewanella haliotis* [39]
IST3	*Shewanella baltica* [39]
IST4	*Vibrio neocaledonicus* [39]
IST5	*Acinetobacter johnsonii* [39]
	*Acinetobacter tjernbergiae* [39]
IST6	*Oceanisphaera donghaensis* [39]
Zuoying Strains	Microorganism
ISZ1	*Vibrio alginolyticus*
ISZ2	*Vibrio harveyi*
	*Vibrio natriegens*
ISZ3	*Vibrio algnolyticus*
ISZ4	*Shewanella algae*
ISZ5	*Oceanimonas baumannii*.

**Table 2:** Elemental composition ratio on the surfaces of 304 SS corresponding to the Tamsui and Zuoying inocula.

**Tamsui**	**Element (wt %)**	**Fe**	**Cr**	**Ni**	**O**	**S**	**P**
12 h	Spectrum 1	74.36	21.11	4.53			
	Spectrum 2	62.39	19.48	8.32	7.78	2.03	
	Spectrum 3	47.40	15.83	5.81	25.91	5.06	
336 h	Spectrum 4	71.87	21.61	6.52			
	Spectrum 5	35.51	11.20	1.83	36.13	15.33	
	Spectrum 6	41.65	7.31	1.54	31.05	18.45	
**Zuoying**	**Element (wt %)**	**Fe**	**Cr**	**Ni**	**O**	**S**	**P**
12 h	Spectrum 7	70.61	21.57	7.82			
	Spectrum 8	54.78	16.99	3.91	17.02	1.28	6.03
	Spectrum 9	61.36	17.50	5.10	12.48	0.70	2.87
336 h	Spectrum 10	74.81	18.05	7.14			
	Spectrum 11	59.86	18.91	8.04	10.10	3.08	
	Spectrum 12	53.64	13.42	3.68	22.12	7.14	

## Data Availability

Data sharing is not applicable.
